# Selective H3 Antagonist (ABT-239) Differentially Modifies Cognitive Function Under the Impact of Restraint Stress

**DOI:** 10.3389/fnsys.2020.614810

**Published:** 2021-02-02

**Authors:** Emil Trofimiuk, Przemysław Wielgat, Halina Car

**Affiliations:** Department of Clinical Pharmacology, Medical University of Bialystok, Bialystok, Poland

**Keywords:** rat, learning and memory (neurosciences), H3R antagonist, restraint stress, Morris water maze, Barnes maze

## Abstract

**Background**: A considerable number of competitive antagonists/inverse agonists of histamine H3 receptor (H3R) have progressed to clinical assessment, with pitolisant approved for the treatment of narcolepsy. H3R, highly expressed in the CNS, is regarded as a relevant target in CNS disorders. At the same time, new compounds including ABT-239 H3R antagonist (ABT; benzonitrile, 4-[2-[2-[(2R)-2-methyl-1-pyrrolidinyl]ethyl]-5-benzofuranyl]-) are continually being tested. The study aimed to test ABT-239 as a prophylactic agent in stress-induced memory impairments.

**Methods**: Stressed and non-stressed rats were pre-treated with ABT-239 and subsequently subjected to several behavioral tests aimed at assessing the animals’ working and spatial reference memory [Morris water maze (MWM), Barnes maze (BM)], assessing the locomotor function and anxiety-like behavior [Open field (OF), elevated “plus” maze—EPM].

**Results**: Chronically stressed rats displayed a significant decline in spatial (working and reference) memory. In the MWM test, we observed an improvement in spatial reference memory in stressed animals and a positive after ABT-239 pre-treatment. In the BM test, the effect of ABT-239 administration on spatial memory changed in successive attempts, from negative initially to favorable in subsequent attempts, and negative in the last trial of the test in the control group of rats. However, a beneficial effect is noted in the group of stressed animals, which remained throughout the entire testing period.

**Conclusions**: Presented findings demonstrate that ABT-239 shows the potential to abolish or prevent restraint stress-induced spatial memory impairments and cognitive deficits. However, in conditions of appetitive modulation, it could increase damage to memory (unstressed animals).

## Introduction

Histamine axons originate from a single source, the tuberomammillary nucleus (TMN) of the hypothalamus, to innervate almost all brain regions. This feature is congruous with the role played by histamine in a host of physiological processes, including the regulation of appetite and body temperature, pain perception, sleep-wake cycle, and cognitive processes.

Histamine has been shown to play an important role in the process of ontogenesis and brain development, including neuronal differentiation (Molina-Hernandez et al., 2012; Rodriguez-Martinez et al., 2012). Therefore, it seems probable and appealing that histaminergic drugs can be useful in stimulating neurogenesis in adults. The pharmacological evaluation of H3R antagonists/inverse agonists, including thioperamide, has revealed their promnesic properties, i.e., enhanced learning ability by improving memory consolidation (Charlier et al., 2013). Literature data demonstrate that these compounds are comparatively safe in animal models (Bajda et al., 2020). It should also be noted that limited peripheral H3R expression is likely to reduce the potential for non-central nervous system side effects that may be associated with the H3 receptor. Among the wide range of H3R antagonists tested to date, ABT-239 and A-431404 have been shown to reduce cognitive deficits induced by ketamine and MK-801, demonstrating a better pro-cognitive effect compared to standard antidepressants drugs (Brown et al., 2013).

The histamine H3R is one of four histamine receptor subtypes classified as the G protein-coupled receptor (GPCRs) family. The human and rat brain regions that widely express H3Rs are those involved in cognition, namely the cerebral cortex, hippocampal formations, striatum, and hypothalamus (Esbenshade et al., 2008). Furthermore, these receptors can control neuronal activity, as observed in histaminergic neurons emerging from the hypothalamus, and the ability to regulate the release of neurotransmitters at the synaptic level in brain areas relating to cognition. Our study focused on ABT-239, a selective, nonimidazole H3R antagonist with high affinity for rat (*pK*_(i)_ = 8.9) and human (*pK*_(i)_ = 9.5) H3Rs, due to literature reports about its pro-cognitive activity (Savage et al., 2010; Munari et al., 2013).

Previously published preclinical data suggest that H3R antagonists exhibit a characteristic profile due to their positive impact on memory improvement (Sadek et al., 2016). A significant link between histamine, H3R, stress, and possibly, cognition has also been established. Histamine release is a sensitive indicator of the stress response and a variety of stress stimuli strongly activate histamine neurons in the tuberomammillary nucleus (Taylor and Snyder, 1971; Brown and Haas, 1999). It has been demonstrated that blockade of brain H3R by selective antagonists results in augmented release and synthesis of histamine and, consequently, the altered release of other important neurotransmitters (ACh, DA, NA, and 5-HT) which play a significant role in cognitive processes and whose levels are disturbed by exposure to chronic stress. Therefore, it would be worth using this interrelationship of H3R to explore the possibility of interference in the pathomechanism of restraint stress regarding complex cognitive functions.

The formation, processing, and use of spatial memory are one of the most important functions of the central nervous system. The performance of relatively simple tasks requires the use of various sources of information—sensory information from one’s surroundings and stored knowledge. The combination of these processes constitutes working memory. It is involved in the temporary storage and coordination of information on relationships occurring in a specific situation and useful only in this situation (Baddeley, 1986). As far as cognition in rats is concerned, working memories are based on events from a specific trial and reference memories are created in the course of repeated trials from the unchanging conditions of a task (Hoing, 1978). Reference memory is long-term memory which represents knowledge regarding the aspects of a task that remain constant between trials and requires memory consolidation involving specific mechanisms. In a spatial task, it mimics two aspects of episodic memory, namely the “what” (content) and “where” (place) dimensions of an event. Working memory is short-term and disappears if it is no longer needed. It is useful only within each trial and does not need to be consolidated for use in future trials. On the other hand, reference memory is long-term memory as it has undergone consolidation.

Our previous studies (Trofimiuk and Braszko, 2014, 2015) revealed that a single administration of an H3R antagonist (ciproxifan) was effective in protecting and improving memory against stress-induced impairments. Therefore, we decided to test the effects of long-term administration of ABT-239, in parallel with exposure to stress, to see if this form of pharmacotherapy will be effective, we use an animal model of chronic restraint stress to test the effectiveness of ABT-239 in preventing/alleviating memory impairment, particularly spatial working and reference memory, under various test conditions.

In our study, we wanted to extract the subtle specificity of the impact of restraint stress and the H3R antagonist as a potent preventive agent on individual memory components and observe the possible dependence on the type of motivation (aversive/appetitive) and stress levels generated the test itself. Due to differences in motivation revealed in our previous studies (Trofimiuk and Braszko, 2014, 2015), which may be relevant to the individualization of therapy, we decided to include this aspect in the current investigation.

## Materials and Methods

### Animals

Sixty-five (6 weeks old) male Wistar rats [Crl: WI (Han) purchased from a certified Laboratory Animal House (Brwinow, Poland)], weighing 140–150 g at the start of the study, were used. They were maintained in a temperature (23°C) and humidity (50–60%) controlled vivarium in groups of three or five in a constant 12/12 h light/dark cycle (lights on at 7:00 AM) with free access to standard lab chow and tap water.

Experimental procedures (Supplementary Datasheet, Timeline table 1) were conducted according to the European Directive, signed on 22 September 2010 (63/2010/EU), and were approved by the Local Ethics Committee for Animal Experimentation.

### Drugs

ABT-239 [ABT; Benzonitrile, 4-[2-[2-[(2R)-2-methyl-1-pyrrolidinyl]ethyl]-5-benzofuranyl], MedChem Tronica, Sweden] was used in all experiments at a dose of 3 mg (Munari et al., 2013) diluted in 1 ml of 0.9% NaCl per kilogram of body weight. It was administered subcutaneously (*s.c*.) every day at 8.00 AM for 21 days. Control rats received 0.9% NaCl in the same volume of 1 ml kg^−1^ for 21 days, every day. Animals were randomly divided into four groups: group 1 consisting of 17 rats receiving 0.9% NaCl s.c. (Control); group 2 comprising 16 rats receiving 3 mg kg^−1^ ABT-239 s.c. (Control + ABT); group 3 consisting of 16 rats receiving 0.9% NaCl s.c. (Stress) which were subsequently subjected to the stress procedure described below; and group 4 comprising 16 rats receiving 3 mg kg^−1^ ABT-239 s.c. which were subsequently subjected to the stress procedure described below (Stress + ABT; see the Supplementary Datasheet, Timeline figure). Particular groups constituted the control for the administered H3R antagonist (ABT-239) and stress so that the impact of specific interventions could be isolated.

### Stress Procedure

Four groups of animals (2 × 8 rats and 2 × 8 rats) were subjected to chronic restraint stress (Avital et al., 2001), 2 h daily for 21 days. The restraint was imposed during the light cycle from 9:00 to 11:00. The animals fitted tightly into the restrainers and were unable to move or turn around. At the same time, unstressed control rats were briefly handled and returned to their cages.

### Behavioral Tests

#### Open Field

Following ABT-239 pre-treatment and completion of the stress procedure, the 33 animals were subjected to the open field (OF) test to assess the psychomotor and musculoskeletal aspects of their performance. Locomotor exploratory activity was measured in an OF (Braszko et al., 1987). A single rat was placed in the center and, following 1 min of acclimatization, crossings, rearings, and bar approaches were counted manually for 5 min.

#### Elevated Plus-Maze

Anxiety-like behavior was evaluated the day after OF performance assessment in the EPM. The arms were arranged so that the open and closed arms were opposite to each other. The 33 rats were placed sequentially in a pre-test arena for 5 min before exposure to the maze. This step allowed the facilitation of exploratory behavior. Immediately after that, the rats were placed in the center of the EPM facing one of the open arms. During the 5-min test period, the number of entries into the open and closed arms and the time spent in the open and closed arms were recorded. An entry was defined as “all four feet in one arm.” An increase in open arm entries and the time spent in open arms was interpreted as indicative of potential anxiolytic activity.

#### Morris Water Maze

The Morris water maze (MWM) task was performed on three consecutive days (Morris et al., 1990), following a period of 3 weeks during which the animals were subjected to stress and were administered ABT-239 treatment. It was conducted during the light period, approximately between 08:00 and 15:00.

On the first day of testing, the animals (32 rats) in the first trial were brought into the testing suite and allowed to find a visible escape platform (9 cm diameter, 2 cm above the water surface, equidistant from the sidewall and the center of the pool), to determine if the administered treatment affected their ability to swim or learn the water maze task. The platform provided the only escape route from the water. The rat was placed on the platform for 15 s for orientation. Following that, it was put in the water, facing the wall of the pool, at the starting position, which was the same for all the studied animals. The basic parameter was the time it took the rat to find the visible escape platform. A rat that failed to reach the platform was given a latency score of 120 s.

Two hours after being tested with a visible platform, the animals were subjected to a test with a hidden platform. In this test, the position of the escape platform (9 cm in diameter, 1.5 cm below the water surface, equidistant from the sidewall and the center of the pool) was changed daily in the pseudo-random sequence. Four different starting points were equally spaced around the perimeter of the pool. On each test day, three starting points (excluding the one in the quadrant containing the platform) were used once in a pseudo-random sequence (i.e., each test commenced at a different starting point). The rat was placed in the water at one of the three starting points, facing the wall of the pool. If the animal failed to find the escape platform within 120 s, it was placed on it for 15 s. The rat that failed to reach the platform was given a latency score of 120 s. The inter-trial interval was 10 min. The animals were trained on three consecutive days, with each animal subjected to one session of three trials per day. Spatial working memory was assessed by the mean of performance results from nine trials and was also analyzed as the mean savings ratio. The mean savings ratio (Glenn and Mumby, 1998) was calculated by representing the second trial latency as a proportion of the total first two trial times per session (i.e., second trial latency/first trial latency + second trial latency). The smaller the saving ratios, the better the retention of the platform position, and the better the working memory.

The border zone was defined as an outer annulus spacing 12 cm from the tank walls. The time spent by the rats in the periphery of the pool was defined as thigmotactic behavior, the tendency to remain close to vertical surfaces.

#### Barnes Maze

The Barnes maze (BM) apparatus is composed of a circular platform (122 cm in diameter) with 18 circular holes (9.5 cm in diameter) evenly spaced around the perimeter (Barnes, 1979; Trofimiuk et al., 2019).

On the first day of training, 33 rats (the same animals that underwent the OP and EPM tests) were brought into the testing suite and allowed to enter the goal box through one of the holes in the maze. Also, some food (pellet of standard lab chow) was placed in the goal box in each trial as a positive stimulus. Once the animal entered the hole, a black Plexiglas cover was placed over the hole to prevent escape. The animal was allowed four minutes for habituation to the goal box after the completion of the first trial. Next, the animal was placed for 30 s in a round, non-transparent holding box (20 cm in diameter, 30 cm high) which was positioned in the center of the maze. The holding box was then removed, a timer set and the investigator moved behind the curtain used only to hide the tester.

The task of escaping from the maze was considered accomplished when all four feet of the animal were in the goal box. Following the successful location of the goal box, the animals were allowed to stay there for 60 s. A maximum of 240 s was allowed for each trial and if an animal did not locate the goal box within this time, it was removed from the maze and placed in the goal box for 60 s.

Two parameters were recorded during each trial. The first was latency to find the goal box and the second the number of errors committed by each animal. An error was defined as a head poke or exploration of any hole other than the hole above the goal box and including perseverant investigations of the same hole.

Spatial working memory was assessed based on the mean of performance results from six trials and was also analyzed as the mean savings ratio. The mean savings ratio (Glenn and Mumby, 1998) was calculated by representing the second trial latency as a proportion of total trial time for the session (i.e., second trial latency/first trial latency + second trial latency). The smaller the savings ratios, the better the retention of escape hole position and the better the working memory.

### Statistical Analysis

Data are presented as means ± standard error of the mean (SEM). A two-way analysis of variance [ANOVA II; treatment × days (trials)] with repeated measures, followed by the *post hoc* Newman–Keuls test for multiple comparisons, was used for the assessment of latency to escape in MWM and BM. One-way analysis of variance (ANOVA), followed by the Bonferroni correction for the comparison of selected groups, was applied to calculate the mean performance of rats in the OF, EPM, MWM, and BM. Statistical significance was set at *p* < 0.05.

## Results

### Effects of ABT-239 Pretreatment on the Locomotor and Exploratory Activity of Rats in the OF

The ANOVA of the results obtained in the OF (Supplementary Figure 1) did not yield any statistically significant differences in the numbers of crossings *F*_(3,31)_ = 2.792 (*p* > 0.05), rearings *F*_(3,31)_ = 1.037 (*p* > 0.05) and bar approaches *F*_(3,31)_ = 0.7817 (*p* > 0.05). This indicates that the treatment and procedures to which the rats in our study were subjected did not affect any aspect of their locomotor activity.

### Effects of ABT-239 Pretreatment on Anxiety-Like Behavior in the EPM

The ANOVA of the results obtained in the EPM test did not yield any statistically significant differences in the time spent by rats in the open arms *F*_(3,31)_ = 0.9463 (*p* > 0.05) and in the numbers of open arm entries *F*_(3,31)_ = 0.08331 (*p* > 0.05; Supplementary Figure 2). This demonstrates that the treatment and procedures to which the rats in our study were subjected did not have a significant impact on the emotional aspect (anxiety) of their psychomotor performance.

### Effects of Stress and ABT-239 on Spatial Memory in the MWM

The ANOVA of latencies to reach the visible platform in the MWM test did not yield statistically significant differences between the groups *F*_(3,31)_ = 0.3806 (*p* > 0.05; Supplementary Figure 3). This indicates that the treatment and procedures to which the rats in our study were subjected did not have a significant impact on their psychomotor performance.

**Figure 1:**
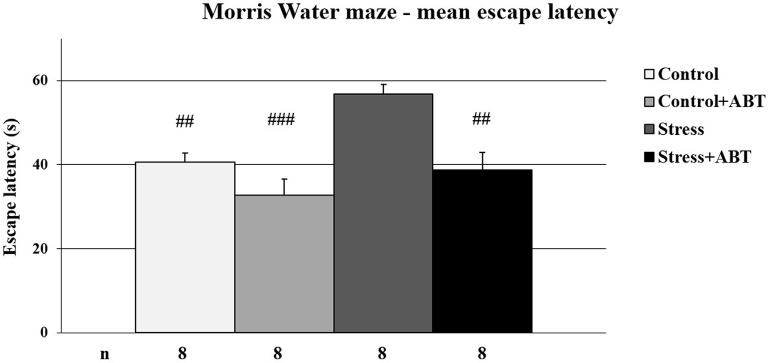
Effects of chronic stress and long-term ABT-239 pre-treatment on performance in spatial memory test of the Morris water maze (MWM) task. Each column represents the mean ± SEM of nine trials (three trials per day for 3 days) obtained from n rats indicated at the bottom of the figure. The three groups demonstrated significantly different results in latencies of reaching submerged platform in the water maze in comparison with stress: ^##^*p* < 0.01 vs. Control, *p* < 0.01 vs. Stress + ABT and ^###^*p* < 0.001 vs. Control + ABT.

The one-way ANOVA of mean latencies to reach the platform in the MWM test yielded statistically significant differences between the groups *F*_(3,31)_ = 10.072 (*p* < 0.001; Figure 1). *Post-hoc* comparisons between preselected pairs with the Bonferroni test revealed that stressed rats reached the escape platform significantly later in comparison to the control animals (*p* < 0.01), and the control rats pre-treated with ABT-239 (*p* < 0.001), as well as stressed animals pre-treated with ABT-239 (*p* < 0.01). These results demonstrate the negative impact of restraint stress on spatial memory and a positive impact of ABT-239 on spatial memory in stressed rats.

**Figure 2:**
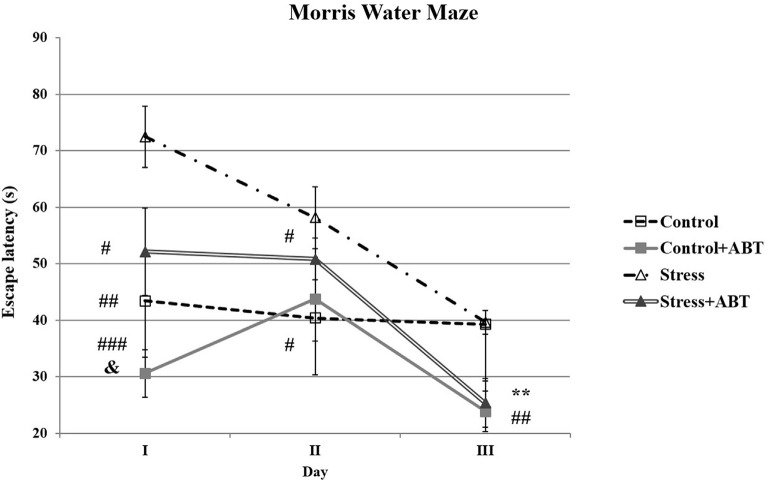
Effects of chronic stress and long-term ABT-239 pre-treatment on performance in spatial memory test of the Morris water maze (MWM) task. Each point of the graph represents the mean ± SEM of three trials (three trials per day for 3 days) obtained from *n* rats indicated at the bottom of the figure. On day I, three groups of rats demonstrated significantly different results in comparison with stress: ^#^*p* < 0.05 vs. Stress + ABT, ^##^*p* < 0.01 vs. Control and ^###^*p* < 0.001 vs. Control + ABT. Statistically significant differences were also in ^&^*p* < 0.05 Control + ABT vs. Stress + ABT. On day II, significantly different results were obtained: Control vs. Stress (^#^*p* < 0.05) and Stress vs. Stress + ABT (^#^*p* < 0.05). On day III, statistically significant differences were: Control vs. Control + ABT and Control vs. Stress + ABT (**p < 0.01), as well as Stress vs. Stress + ABT and Stress vs. Control + ABT (^##^*p* < 0.01).

The ANOVA II of latencies to reach the platform in the water maze revealed a significant effect of the administered treatment (*F*_(3,31)_ = 10, 023; *p* < 0.001) and a significant day effect (*F*_(2,6)_ = 17.861; *p* < 0.001) and a significant treatments × days interaction (*F*_(6,56)_ = 3.834; *p* < 0.01; Figure 2). This suggests that all the studied rats learned the task efficiently and specific treatment and time effects were detected.

Interesting relationships were observed on particular days of training (Figure 2). We found that on the first day *F*_(3,31)_ = 10.432 (*p* < 0.001), stressed rats performed worse at finding the escape platform than the control animals and demonstrated poorer spatial working and reference memory (*p* < 0.01) in comparison to control rats pre-treated with ABT-239 (*p* < 0.001) as well as stressed rats pre-treated with ABT-239 (*p* < 0.05). Statistically significant differences were also observed between the Stress + ABT group in comparison to the Control + ABT group (*p* < 0.05). Therefore, the effect of ABT-239 administration on the improvement of spatial memory could be observed, particularly in stressed animals. The impact on control animals was also noticeable, but it was not statistically significant. Thus, on the first day, we observed a clear mitigation of the negative impact of stress. On the second day of training *F*_(3,31)_ = 2.880 (*p* < 0.05), the detrimental impact of stress on control animals was still significant (*p* < 0.05). Similarly, the beneficial effect of ABT-239 was also maintained regarding alleviating the negative impact of stress (*p* < 0.05). On the third day of training in the MWM *F*_(3,31)_ = 6.339 (*p* < 0.01), the adverse effect of stress in the studied groups in comparison to control animals disappeared (*p* > 0.05). However, the beneficial impact of the administered ABT-239 remained, both in stressed (Stress vs. Stress + ABT, *p* < 0.01; Stress + ABT vs. Control, *p* < 0.01) and control animals (Control vs. Control + ABT, *p* < 0.01; Control + ABT vs. Stress, *p* < 0.01).

The ANOVA of the calculated mean savings ratios from the MWM test yielded *F*_(3,31)_ = 2.683 (*p* > 0.05), showing no statistically significant differences between the groups (Supplementary Figure 4). The obtained results indicate that the examined factors did not influence the spatial working memory of either stressed or ABT-239 treated animals in the MWM test. Therefore, it can be assumed that the significant differences observed between the groups in escape latency may result from the disturbance of other spatial memory components, including reference memory. Good visible-platform training results coincided with impaired hidden-platform performance, which indicates that motivational/emotional or sensorimotor defects did not contribute significantly to the hidden-platform test deficits.

**Figure 3:**
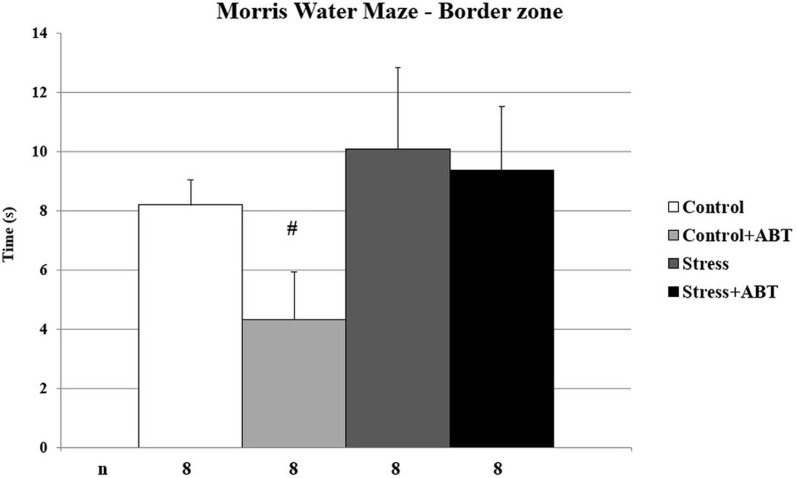
Effects of chronic stress and long-term ABT-239 pre-treatment on border zone latency in MWM task. Each column represents the mean ± SEM of nine trials (three trials per day for 3 days) obtained from *n* rats indicated at the bottom of the figure. Control rats pre-treated with ABT-239 spent significantly less time in the border zone than the stressed group (^#^*p* < 0.05).

As shown in Figure 3, the ANOVA of MWM test results yielded statistically significant differences in the mean time spent in the border zone (BZ) of the maze *F*_(3,31)_ = 1.704 (*p* < 0.05). *Post-hoc* comparisons in preselected pairs with the Bonferroni test revealed that control rats pre-treated with ABT-239 spent significantly less time in the border zone in comparison to the stressed group (*p* < 0.05). This may indicate that ABT-239 had a positive impact on control animals, expressed by an intensified search for the exit. We did not observe this effect in stressed animals.

### Effects of Stress and ABT-239 on the Performance of Rats in the BM Test

**Figure 4:**
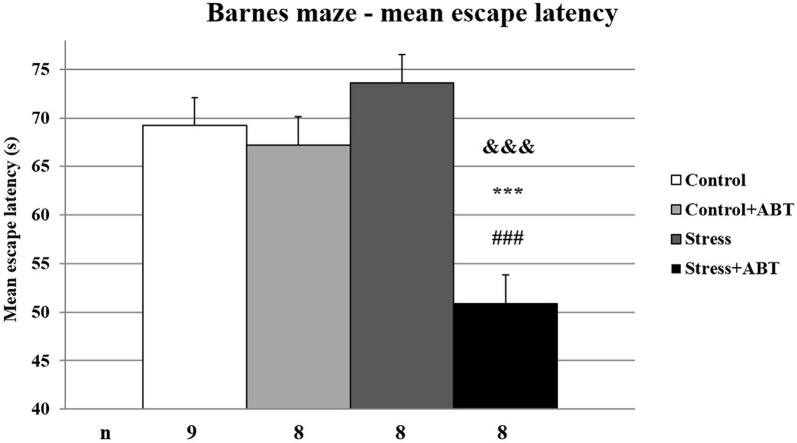
Effects of chronic stress, long-term ABT-239 treatment on mean escape latency in the spatial memory test of the Barnes maze (BM) task. Each column represents the mean ± SEM of six trials (two trials per day for 3 days) obtained from *n* rats indicated at the bottom of the figure. The three groups demonstrated significantly different results in mean latencies of reaching escape hole in the BM in comparison with Stress + ABT: ***p < 0.001 vs. Control, ^###^*p* < 0.001 vs. Stress, ^&&&^*p* < 0.001 vs. Control + ABT.

The ANOVA of mean latencies to enter the escape hole in the BM test yielded *F*_(3,32)_ = 11.364 (*p* < 0.05), showing significant differences between the groups (Figure 4). Time spent searching for the escape hole by rats pre-treated with ABT-239 and stressed animals was significantly shorter in comparison to all other studied groups (*p* < 0.001 vs. Control; *p* < 0.001 vs. Control + ABT; *p* < 0.001 vs. Stress). Mean escape latency analysis is based on hippocampal-dependent spatial working and reference memories. The data obtained after averaging the results indicate that ABT-239 had a particularly favorable impact on stressed animals. This effect was not observed in control animals.

The ANOVA II of latencies to reach the escape hole in the BM revealed a significant impact of the administered treatment (*F*_(3,32)_ = 11.200; *p* < 0.001) and also significant day effect (*F*_(5,15)_ = 86.865; *p* < 0.001) and a significant treatments × days interaction (*F*_(15,145)_ = 7.329; *p* < 0.001). This suggests that all the studied rats learned the task efficiently, and specific treatment and time effects were detected.

**Figure 5:**
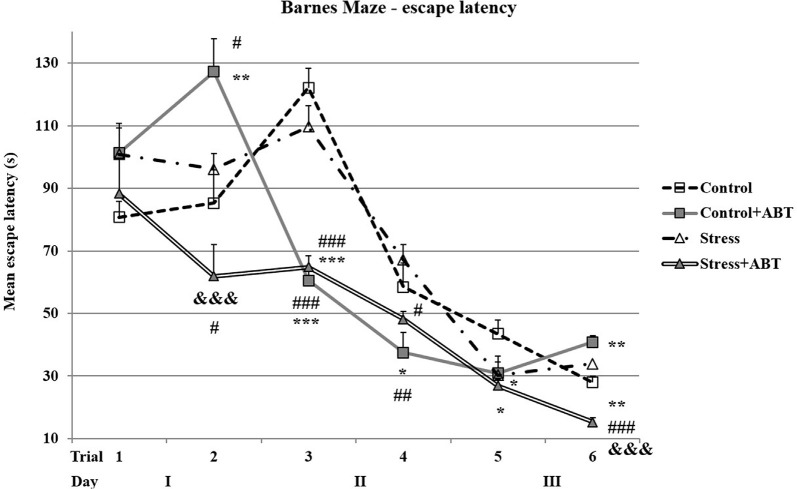
Effects of chronic stress, prolonged ABT-239 pre-treatment on performance in spatial memory test of the BM task. Each point of the graph represents the mean escape latency ± SEM obtained from eight to nine rats (two trials per day). In the first trial, no significantly different results were found. In the second trial, Control rats were significantly different in comparison with Control + ABT (^##^*p* < 0.01) and stressed with: ^#^*p* < 0.05 vs. Control + ABT; ^#^*p* < 0.05 vs. Stress + ABT. Statistically significant differences were also in Stress + ABT vs. Control + ABT (^&&&^ *p* < 0.001). In the third trial, Control rats were significantly different in comparison with Control + ABT (***p < 0.001) and with Stress + ABT (***p < 0.001). Statistically significant differences were also found in stressed rats with Stress + ABT (^###^*p* < 0.001) and with Control + ABT (^###^*p* < 0.001). In the fourth trial, Control rats were significantly different in comparison with Control + ABT (*p < 0.05) and Stressed rats in comparison with Stress + ABT (^#^*p* < 0.05) as well as with Control + ABT (^##^*p* < 0.01). In the fifth trial, the differences were statistically significant in Control rats in comparison with Stress (*p < 0.05) and Stress + ABT (*p < 0.05). In the sixth trial, statistically significant differences were noted in Control vs. Control + ABT (**p < 0.01), Control vs. Stress + ABT (**p < 0.01); Control + ABT vs. Stress + ABT (^&&&^*p* < 0.001), Stress + ABT (^###^ *p* < 0.001) groups.

When analyzing the results obtained on particular days, we observed some interesting trends and phenomena (Figure 5). In the first trial, *post hoc* comparisons in preselected pairs with the Bonferroni test did not reveal any significant differences between the groups (*F*_(3,32)_ = 1.528; *p* > 0.05). The pre-treatment with ABT-239 exerted a detrimental impact on control rats (Control vs. Control + ABT, *p* < 0.01), which was greater than that caused by stress (*p* < 0.05 vs. Stress) and, particularly, the stress group pre-treated with ABT-239 (Control + ABT vs. Stress + ABT, *p* < 0.001). In the group of stressed animals, ABT-239 administration produced a positive effect (*p* < 0.05 Stress vs. Stress + ABT). In the third trial, statistical significance reappeared *F*_(3,32)_ = 41.203 (*p* < 0001). Time spent searching for the escape hole by control rats was significantly longer in comparison to control animals pre-treated with ABT-239 (Control vs. Control + ABT, *p* < 0.001). Stressed rats pre-treated with ABT-239 found the escape hole faster than control rats (Stress + ABT vs. Control, *p* < 0.001) and stressed animals (*p* < 0.001 Stress + ABT vs. Stress) as well as control rats pre-treated with ABT-239 in comparison with stressed animals (Control + ABT vs. Stress, *p* < 0.001). This time, the positive effect of the H3R antagonist administration was evident in both control and stressed animals. In the fourth trial *F*_(3,32)_ = 3.956 (*p* < 0.05) statistical significances were also established. The positive effect of ABT-239 administration was maintained in both control (Control + ABT vs. Control, *p* < 0.05) and stressed animals (Stress + ABT vs. Stress, *p* < 0.05). Besides, the difference in the effect of ABT-239 administration was also significant (Control + ABT vs. Stress; *p* < 0.01). In the fifth trial *F*_(3,32)_ = 2.848 (*p* < 0.05), the only significant changes were observed in control rats (Control vs. Stress, *p* < 0.05; Control vs. Stress + ABT; *p* < 0.05). In the sixth trial *F*_(3,32)_ = 17.102 (*p* < 0.001), the obtained results showed that stressed rats pre-treated with ABT-239 were significantly faster to reach the escape hole in comparison with control rats (Stress + ABT vs. Control; *p* < 0.01) and control animals pre-treated with ABT-239 (Stress + ABT vs. Control + ABT; *p* < 0.001), and even faster in comparison to stressed rats (Stress + ABT vs. Stress; *p* < 0.001). Moreover, the difference in the effect of ABT-239 administration was also significant in control animals (Control vs. Control + ABT; *p* < 0.01).

**Figure 6:**
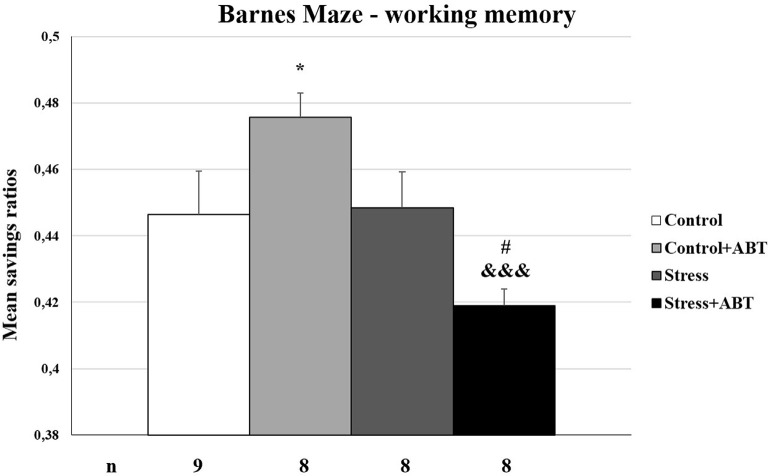
Effects of stress and long-term ABT-239 pre-treatment on working memory expressed in mean savings ratios tested in BM. Columns represent mean savings ratios ± SEM from 6 days of trials obtained from *n* rats indicated at the bottom of the figure. The three groups demonstrated significantly different results in mean savings ratio achieved in the BM Control in comparison with Control + ABT (*p < 0.001), Control + ABT vs. Stress + ABT (^#^*p* < 0.05), and Stress vs. Stress + ABT (^&&&^*p* < 0.001).

The ANOVA of the calculated mean savings ratios from the BM test yielded *F*_(3,32)_ = 5.379 (*p* < 0.001), showing statistically significant differences between the groups (Figure 6). Pre-treatment with ABT-239 exerted a detrimental effect on control rats (Control vs. Control + ABT; *p* < 0.05). A radically different effect occurred in the group of stressed animals, in which the administration of ABT-239 significantly improved working memory (*p* < 0.05; Stress vs. Stress + ABT). Statistically significant differences were also observed in the Stress + ABT group in comparison to the Control + ABT group (*p* < 0.001). Again, the negative effect of ABT-239 administration in the control animal group was seen, stronger than stress (not significantly), and positive in the stressed ones.

**Figure 7:**
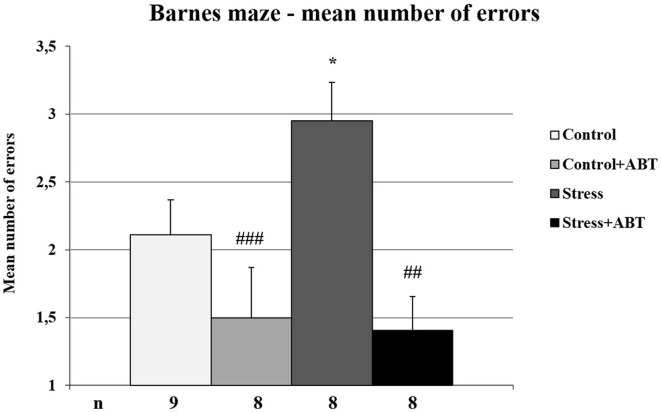
Effects of chronic stress, prolonged ABT pre-treatment on performance in working memory test of the BM task. Each column represents the mean ± SEM number of errors from 12 trials (two trials per day for 6 days) obtained from *n* rats indicated at the bottom of the figure. The three groups demonstrated significantly different results in time to escape in comparison with Stress: vs. Control (*p < 0.05); vs. Control + ABT (^##^*p* < 0.01) and vs. Stress + ABT (^###^*p* < 0.001).

The one-way ANOVA of the mean numbers of errors made by rats in the BM test yielded *F*_(3,32)_ = 5, 703 (*p* < 0.001), showing statistically significant differences between the groups (Figure 7). Further *post hoc* comparisons made with the Bonferroni test revealed a significantly higher number of errors made by stressed rats in comparison to control animals (Stress vs. Control, *p* < 0.05), control rats pre-treated with ABT-239 (Stress vs. Control + ABT; *p* < 0.01), and stressed rats pre-treated with ABT-239 (Stress vs. Stress + ABT; *p* < 0.001).

Therefore, it seems that restraint stress adversely affects this type of memory, particularly working memory, and there is a noticeable positive effect of ABT administration on stressed but not always on control rats.

## Discussion

In this work, we tested the effects of ABT-239 in various behavioral tests. In the MWM test assessing spatial, reference, and working memory we observed the adverse effects of restraint stress. The test requires animals to actively search for an exit (hidden platform). The animal is forced to swim in cold water (aversive motivation). The test itself generates a certain level of stress (Stewart and Morris, 1993), because of a sense of uncertainty and danger, sometimes taking the form of a desperate search for a way out (escape platform). Therefore, even though the location of the platform changed, the animals found it faster and with greater ease in each subsequent trial. Thus, the animals used the memory that had been consolidated, i.e., long-term memory—reference memory. To separate the reference memory share from working memory, we decided to evaluate mean savings ratios.

In the assessment of spatial memory performance, ABT-239 proved to be effective. When we evaluated the average escape latency from 3 days (nine trials), the effect of ABT-239 was significant. In the MWM test, we observed alleviation of the adverse impact of stress on cognitive performance, which was restored to the level displayed by control animals. At the same time, the effect of ABT-239 on control (non-stressed) animals was more profound compared to stressed animals, although it was not statistically significant. However, a detailed analysis of the results obtained on each day of the test revealed the nootropic effect of ABT-239 in control animals. ABT-239 administration showed efficacy and even nootropic effects in the control group, which we did not record with the ciproxifan used in our previous study (Trofimiuk and Braszko, 2015).

The potential beneficial effects of ABT-239 on cognitive function have been described previously (Savage et al., 2010; Varaschin et al., 2010). However, our study revealed that the observed effects relate to specific types of memory. We are the first researchers to show that in aversive conditions ABT-239 improves spatial memory without affecting working memory based on MWM results. Another H3R blocker, ciproxifan, we used in the previous study (Trofimiuk and Braszko, 2015), showed this activity, i.e., it affected working memory. Simultaneously, it transpired that in the group of control animals, pre-treatment with ABT-239 had a positive impact on the strategy of searching for an exit, effectively reducing time spent by the animals in the border zone (thigmotaxis). While the ciproxifan used in the previous study (Trofimiuk and Braszko, 2015) did not show an activity reducing thigmotaxis by itself, ABT-239 does. However, ciproxifan significantly decreased border zone time in the stress-exposed group, which is not seen with ABT-239. Yet, the results of the EPM test, assessing anxiety-like behavior in animals, did not reveal differences between the studied groups in both cases. Therefore, unfavorable test conditions (forced swimming) may translate into increased anxiety which cannot be observed when animals move to a dry surface. It is well known that exposure to stress increases the anxiety of test animals (Shekhar et al., 2005). Interestingly, we did not observe this effect in the group of stressed animals. Hence, it can be assumed that the observed difference was due to disturbances relating to the exit search strategy.

H3R ligands have been the subject of research into the central nervous system and represent a promising approach (Alguacil and Pérez-García, 2003; Bajda et al., 2020). A range of competitive antagonists/inverse agonists have progressed to clinical trials, with pitolisant approved for the treatment of narcolepsy. Given the range of compounds developed and their potential therapeutic indications, we assessed the most promising compound, ABT-239, and evaluated the efficacy of its long-term use in an animal model of chronic restraint stress exposure.

The positive impact of ABT-239 on cognitive function should be seen in augmented release and synthesis of histamine and, consequently, the altered release of other important neurotransmitters such as ACh, DA, NA, and 5-HT (Fox et al., 2005). ABT-239 enhances acetylcholine release in the frontal cortex and hippocampus of an adult rat and increases dopamine release in the frontal cortex (Fox et al., 2005). Moreover, Munari et al. (2013) have shown that ABT-239 increases c-Fos expression dose-dependently in the rat cortex and nucleus basalis magnocellularis, augments acetylcholine, and histamine release from the rat prefrontal cortex. According to other recent studies, ABT-239 also displays appreciable sigma-1 receptor affinity (Riddy et al., 2019). It is worth focusing on this compound since there are reports (Vavers et al., 2019) that sigma-1 receptor modulators may also have a beneficial effect on cognitive function and alleviate the negative impact of stress (antidepressant properties). However, to assess and confirm the participation of this mechanism in the effect observed by us in this work, further studies should be performed.

Studies are indicating the effectiveness of spatial memory modulation by H3 receptors. Namely, S38093, another novel histamine H3R inverse agonist, after acute oral administration, significantly improved working spatial memory in the MWM test and showed nootropic effects in a two-trial object recognition task in rats (Panayi et al., 2017). Another active molecule (ABT-288) administered acutely and sub-acutely improved spatial learning and reference memory in the MWM test, acquisition of a five-trial inhibitory avoidance test, and social recognition memory in rats (Esbenshade et al., 2012).

Another test conducted as part of our study demonstrated that in less stressful conditions such as the BM, where animals are appetitively motivated, the effects of ABT-239 pre-treatment can be different. The effect of ABT-239 administration was noticed only in the group of stressed animals. We detected an obvious improvement in this group of animals compared to both animals exposed to stress and control rats in the context of mean escape latency analysis (hippocampal-dependent reference and spatial working memory). The applied ABT-239 did not deteriorate memory in the control group, as it was in the case of ciproxifan (Trofimiuk and Braszko, 2015), and it significantly improved it in the stressed animal’s group. This speaks in favor of this substance that it has no global negative effect on control animals, and in the group of stressed animals, it improved spatial memory significantly better than controls. As in the MWM test, in the BM test, the reference memory contribution could not be completely excluded in the mean escape latency analysis. Notwithstanding a detailed analysis, which took into consideration the results of individual trials, demonstrated that the administration of ABT-239 to control animals may have adversely affected their performance in the test (performance on two out of six trials of the test was worse than that of the controls), although we also observed a nootropic effect (on the third and fourth trial of the test), which finally balances out. We observed the beneficial effects of ABT-239 administration on test performance by stressed animals. In this group of animals, the improvement concerned both spatial reference and working memory (assessed as the analysis of mean savings ratios). The situation was significantly different in the control group, where we observed a deterioration in working memory (calculated as mean savings ratios) after prolonged treatment with the H3R antagonist, this is somewhat surprising because in our previous study (Trofimiuk and Braszko, 2015) using another H3 receptor blocker, ciproxifan, we did not see such an effect. Moreover, in the group of stressed animals, we observed a significant improvement for control, which was not the case with ciproxifan. The parameter which effectively evaluated working memory in this test was the number of errors made (visiting the same hole again). It is surprising that concerning this parameter (mean number of errors), the obtained results were the opposite. Stressed animals committed a far greater number of errors in comparison to controls. Preventive administration of ABT-239 successfully reduced the number of errors in both the control group and the group stressed animals. Since the differences in the average time taken to find the exit were not significant, except for the stressed animals, this may indicate a better use of time and a painstaking search for the exit (fewer errors). Therefore, the mean savings ratio parameter does not seem to fully reflect the actual situation. Relying on this parameter alone can lead to wrong conclusions. However, it requires checking, preferably on a larger group of animals. It would also be worth exploring the impact of continuous administration of the compound because our research model assumed discontinuation of administration as well as the termination of stress exposure before subjecting animals to behavioral tests. It would be worth testing ABT-239 in another experimental model.

Unfortunately, in addition to the promising results obtained during the use of ABT-239, it should also be mentioned and noted that during the safety assessment carried out on cynomolgus macaques and rats, an adverse effect on the electrical conduction system of the heart, QT prolongation, and a narrow safety margin were shown (Cowart et al., 2005; Hancock, 2006).

To recapitulate, we are the first researchers to demonstrate that the use of ABT-239 effectively prevents cognitive impairment caused by chronic restraint stress. The compound demonstrated broad efficacy in multiple behavioral tests. These types of behavioral tests tap into multiple neurotransmitter systems and multiple cognitive domains and predict that compounds such as ABT-239 would have the potential for clinical efficacy in human cognitive deficiency caused by various factors, in this case, stress-induced. The H3R antagonist used in the study improved both spatial reference memory and working memory, in particular working memory capacity and processing. This seems to be an interesting possibility, but requiring further research, to use H3R antagonists to alleviate memory disturbances caused by stress.

## Data Availability Statement

The raw data supporting the conclusions of this article will be made available by the authors, without undue reservation.

## Ethics Statement

The animal study was reviewed and approved by Local Ethics Committee for Animal Experimentation in Olsztyn.

## Author Contributions

ET, PW, and HC participated in research design and contributed to the writing of the manuscript. ET and PW conducted experiments. ET contributed new reagents and performed data analysis. All authors contributed to the article and approved the submitted version.

## Conflict of Interest

The authors declare that the research was conducted in the absence of any commercial or financial relationships that could be construed as a potential conflict of interest.

## Supplementary Material

SUPPLEMENTARY FIGURE 1 Effects of chronic stress, long term ABT-239 administration on the locomotor activity of rats in the Open field. Each column represents the mean ± SEM of the number of crossings, rearings and bar approaches obtained from n rats indicated at the bottom of the figure.

SUPPLEMENTARY FIGURE 2 Effects of chronic stress and long-term ABT-239 pretreatment on the time spent by rats in, or the number of entries to the open arms of elevated plus-maze. Columns represent means ± SEM of the values obtained from n rats indicated at the bottom of the figure.

SUPPLEMENTARY FIGURE 3 Effects of chronic stress and long-term ABT-239 pre-treatment on locomotor performance in reaching visible platform in Morris water maze task. Each column represents the mean ± SEM of first trial obtained from n rats indicated at the bottom of the figure.

SUPPLEMENTARY FIGURE 4 Effects of stress and long-term ABT-239 pre-treatment on working memory expressed in mean savings ratios tested in water maze. Columns represents mean savings ratios ± SEM from nine trials (three trials per day for three days) obtained from n rats indicated at the bottom of the figure.
