# Supplementary figures and images for: Selective H3 Antagonist (ABT-239) Differentially Modifies Cognitive Function Under the Impact of Restraint Stress
Source: Front Syst Neurosci. 2021 Feb 2;14:614810. doi: 10.3389/fnsys.2020.614810 (PMC7884464; doi:10.3389/fnsys.2020.614810)

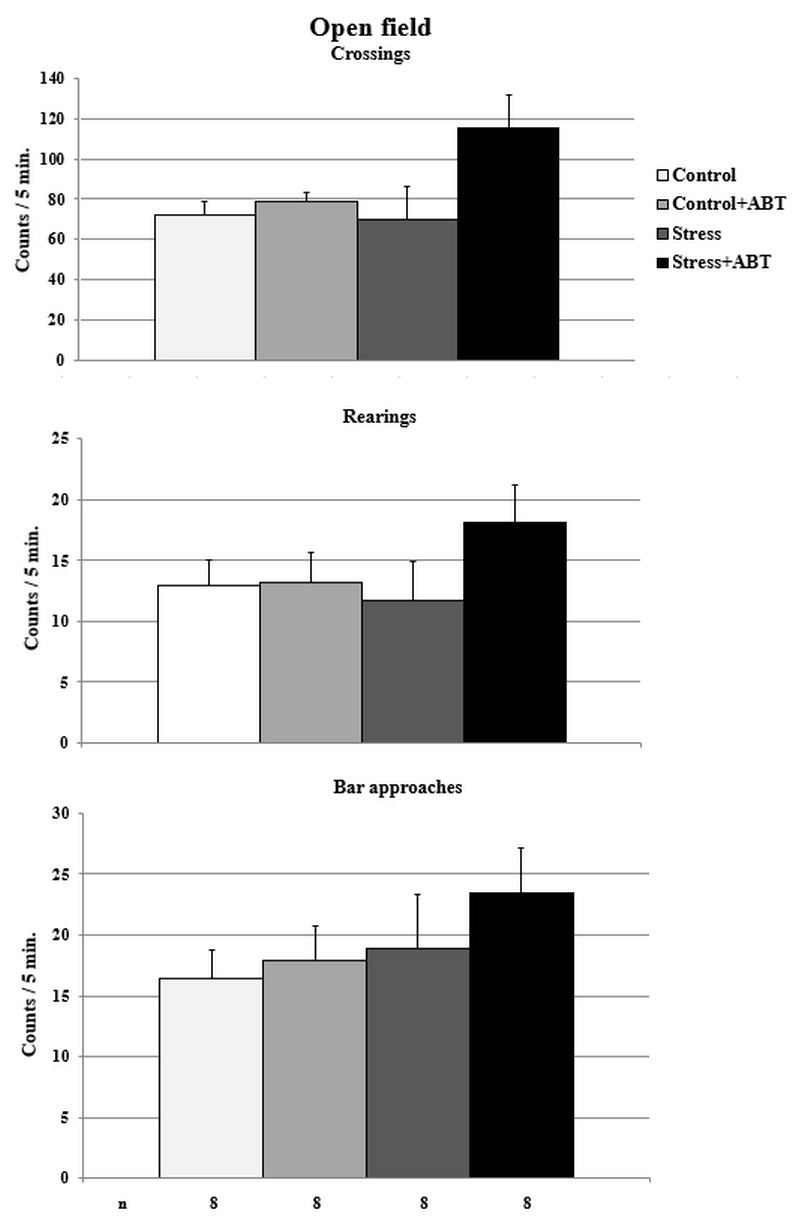

Supplement: SUPPLEMENTARY FIGURE 1 — Effects of chronic stress, long term ABT-239 administration on the locomotor activity of rats in the Open field. Each column represents the mean ± SEM of the number of crossings, rearings and bar approaches obtained from n rats indicated at the bottom of the figure. [file Image_1.JPEG]

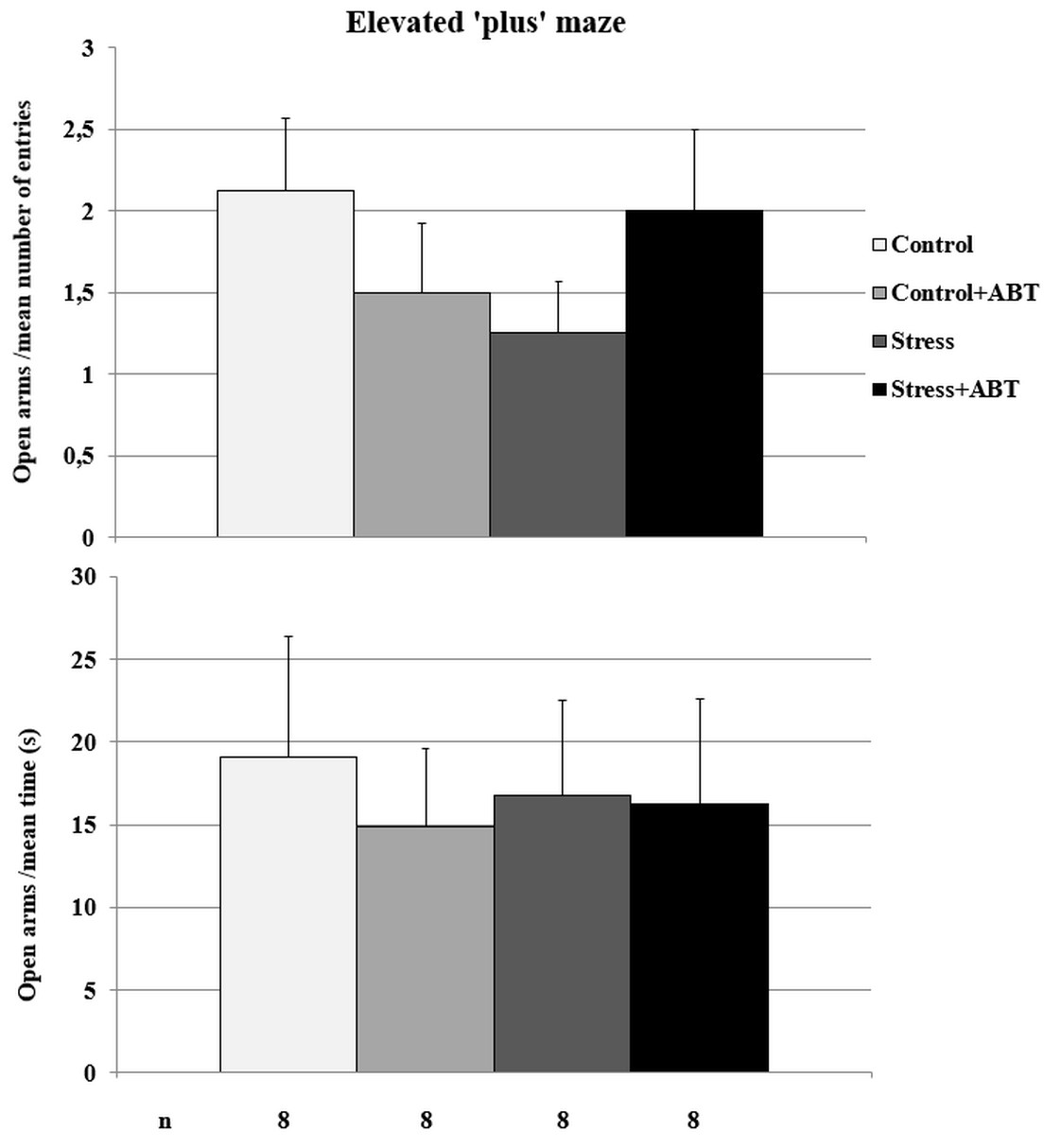

Supplement: SUPPLEMENTARY FIGURE 2 — Effects of chronic stress and long-term ABT-239 pretreatment on the time spent by rats in, or the number of entries to the open arms of elevated plus-maze. Columns represent means ± SEM of the values obtained from n rats indicated at the bottom of the figure. [file Image_2.JPEG]

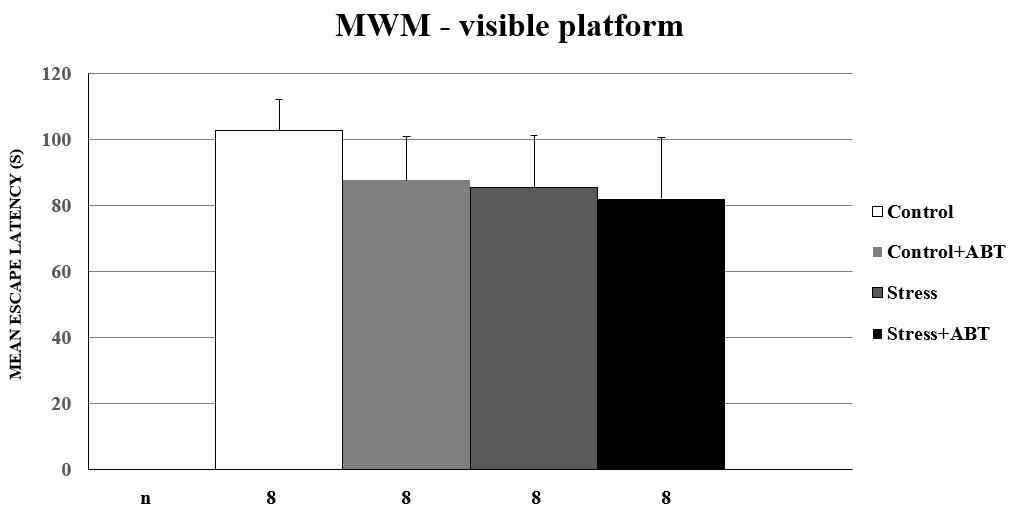

Supplement: SUPPLEMENTARY FIGURE 3 — Effects of chronic stress and long-term ABT-239 pre-treatment on locomotor performance in reaching visible platform in Morris water maze task. Each column represents the mean ± SEM of first trial obtained from n rats indicated at the bottom of the figure. [file Image_3.JPEG]

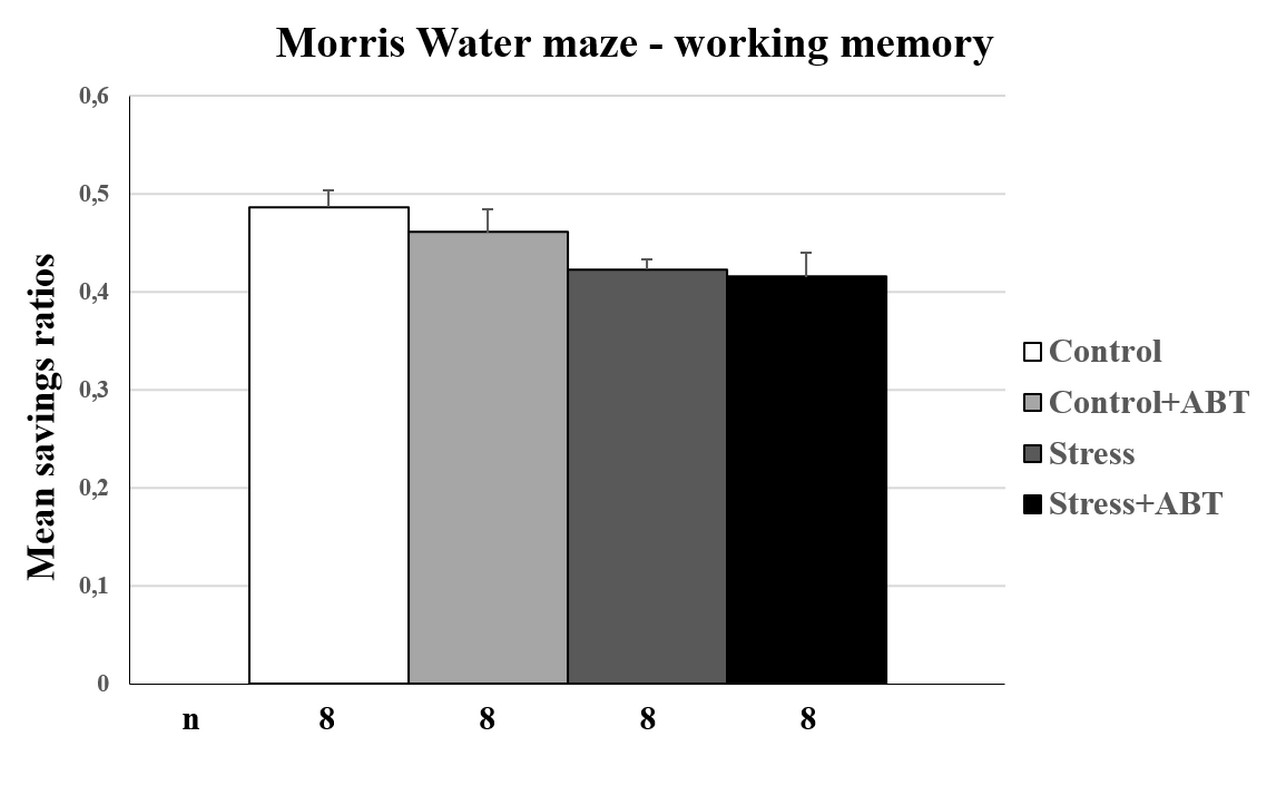

Supplement: SUPPLEMENTARY FIGURE 4 — Effects of stress and long-term ABT-239 pre-treatment on working memory expressed in mean savings ratios tested in water maze. Columns represents mean savings ratios ± SEM from nine trials (three trials per day for three days) obtained from n rats indicated at the bottom of the figure. [file Image_4.JPEG]
