# Supplementary material for: Selective H3 Antagonist (ABT-239) Differentially Modifies Cognitive Function Under the Impact of Restraint Stress
Source: Front Syst Neurosci. 2021 Feb 2;14:614810. doi: 10.3389/fnsys.2020.614810 (PMC7884464; doi:10.3389/fnsys.2020.614810)

Timeline diagram: Flow diagram of the experiment. Subsequent procedures were carried out on two different groups of animals. The procedures of ABT administration (or vehicle) and stress (or handling) were common. In the first group of animals after 20 days of administration procedure an OF test was carried out one hour after completion of the procedures. In the same group of animals, the next day and before BM, EPM was performed. In the second group of animals MWM was performed.

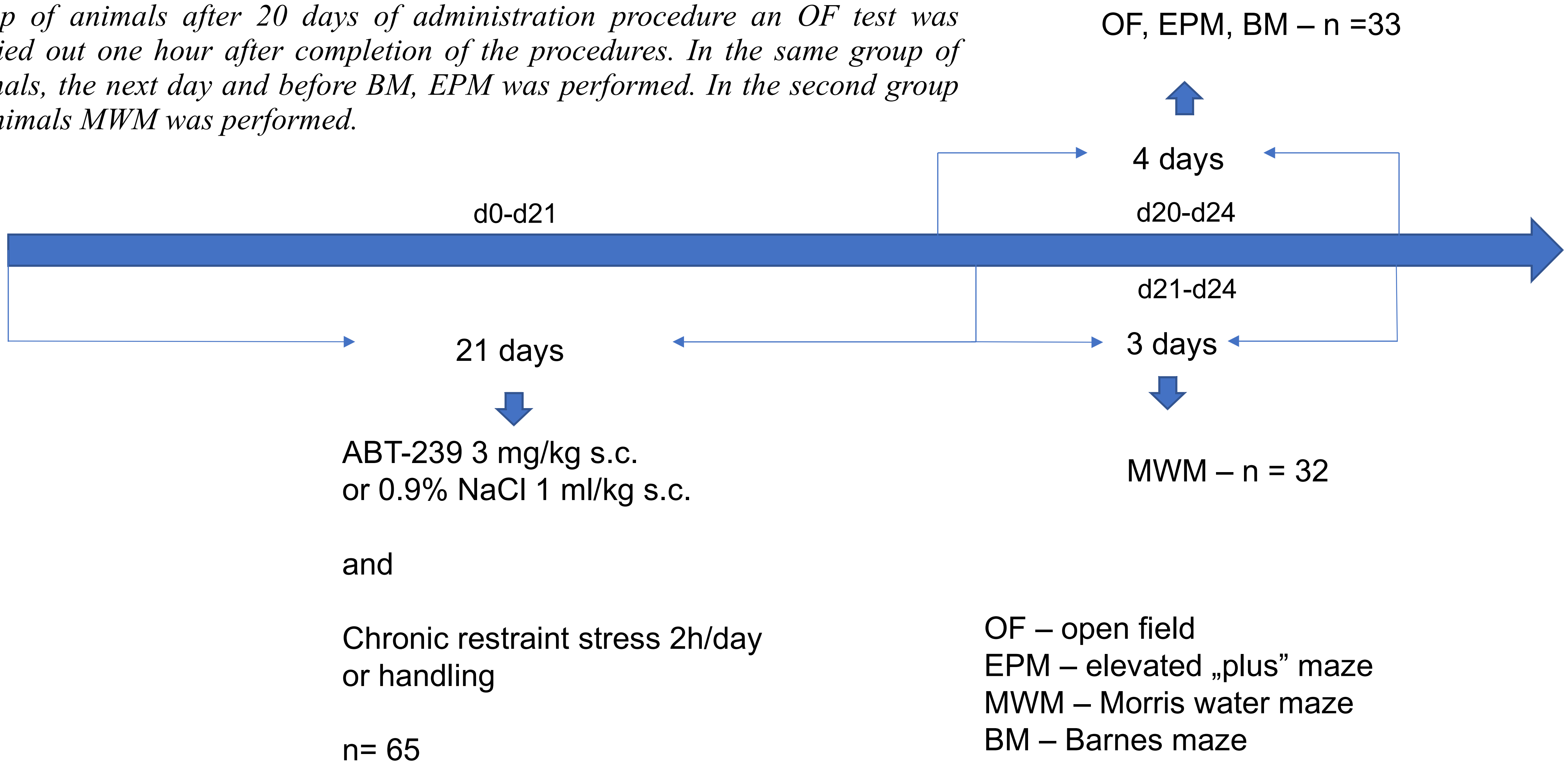

Supplement: Supplementary file 5 [file Data_Sheet_1.PDF]
